# Dementia Incidence, Burden and Cost of Care: A Filipino Community-Based Study

**DOI:** 10.3389/fpubh.2021.628700

**Published:** 2021-05-14

**Authors:** Jacqueline Dominguez, Leo Jiloca, Krizelle Cleo Fowler, Ma. Fe De Guzman, Jhozel Kim Dominguez-Awao, Boots Natividad, Jeffrey Domingo, Jayvee Dyne Dominguez, Macario Reandelar, Antonio Ligsay, Jeryl Ritzi Yu, Stephen Aichele, Thien Kieu Thi Phung

**Affiliations:** ^1^Institute for Neurosciences, St. Luke's Medical Center, Quezon City, Philippines; ^2^Institute for Dementia Care Asia, Quezon City, Philippines; ^3^Geriatric Center, St. Luke's Medical Center, Quezon City, Philippines; ^4^Research and Biotechnology Division, St. Luke's Medical Center, Quezon City, Philippines; ^5^Department of Internal Medicine, St. Louis University Hospital, Baguio, Philippines; ^6^Section of Clinical Research, St. Luke's Medical Center - College of Medicine, Quezon City, Philippines; ^7^Department of Human Development and Family Studies, Colorado State University, Fort Collins, CO, United States; ^8^Danish Dementia Research Center, Rigshospitalet, University of Copenhagen, Copenhagen, Denmark

**Keywords:** dementia incidence, cost of care, DALY, LMIC, Filipino

## Abstract

**Background:** In the midst of competing priorities and limited resources in low-middle-income countries (LMIC), convincing epidemiological evidence is critical for urging governments to develop national dementia plans. The majority of primary epidemiological studies on dementia are from high income countries (HIC). Implications for developing countries are typically extrapolated from these outcomes through modeling, meta-analyses, and systematic reviews. In this study, we directly assessed the incidence of dementia, disability adjusted life years (DALYs), and cost of care among community-dwelling Filipino elderly.

**Methods:** This was a follow-up study of the prospective cohort Marikina Memory Ageing Project (MMAP). Baseline assessment was performed in 2011–2012, and follow-up was done in 2015–2016 (*N* = 748 at follow-up). Incident dementia was determined. Disease burden was computed using the incidence rates and DALYs. Both indirect and direct (medical and non-medical) costs of dementia care were computed.

**Results:** The crude incidence rate was 16 (CI: 13–20) cases per 1,000 person-years (pyr) with 17 (CI: 12–21) per 1,000 pyr for females and 14 (CI: 9–21) per 1,000 pyr for males. Based on this incidence, we project an estimation of 220,632 new cases in 2030, 295,066 in 2040, and 378,461 in 2050. Disease burden was at 2,876 DALYsper 100,000 persons. The economic burden per patient was around Php 196,000 annually (i.e., ~4,070 USD, or 36.7% of average family annual income in the Philippines). The majority (86.29%) of this care expense was indirect cost attributed to estimated lost potential earning of unpaid family caregivers whereas direct medical cost accounted for only 13.48%.

**Conclusions:** We provide the first Filipino community-based data on the incidence of dementia, DALYs, and cost of care to reflect the epidemiologic and economic impact of disease. The findings of this study serve to guide the development of a national dementia plan.

## Introduction

In the midst of competing priorities and limited resources, low-middle-income countries (LMIC) are projected to have the largest rise in dementia prevalence by 2050 (1, 2). Convincing epidemiological evidence is critical for urging governments to develop national plans to address the dementia epidemic. Large datasets like the 10/66 Dementia Research Group surveys have provided robust data on dementia occurrence in developing countries (3). The estimated prevalence of dementia in Southeast Asia is expected to increase to 12.09 million (236%) by 2050 (1). However, epidemiological studies are lacking in Southeast Asian LMIC countries. The Filipino aging population comprises ~7% of the country's total population in 2015 and is projected to increase by 11% in 2025 and 16% in 2045 (4). In the Philippines, the estimated number of elderly (60 years and older) will more than double from 9.5 million in 2020 to 19.7 million in 2040 (5). The extent of dementia in the Philippines however has not yet been estimated, and data are lacking. In 2011, we conducted the Marikina Memory and Aging Project (MMAP), a population-based study among Filipino older adults aged 60 years and over which yielded a dementia prevalence of 10.6%. In the study, approximately a quarter (23.2%) were identified to have mild cognitive impairment (MCI) along with an alarmingly high prevalence of cardiovascular risk factors among Filipino older adult men and women, further increasing the risk for developing dementia (6). However, this is not adequately addressed, and dementia is currently not recognized as a major public health issue in the Philippines. This makes the case for establishing epidemiological studies that can increase awareness of the magnitude of the problem and guide government policy in allocating resources to dementia care and prevention, which is especially critical in low-resource health care settings. More specifically, it is essential to have data on burden and cost of care to inform the government of the economic impact of the disease on extended families, who in the Philippines remain the cornerstone of dementia care and of the society. In the current study, we determined the incidence of dementia, disability adjusted life years, and cost of care of dementia among community-dwelling Filipino elderly.

## Methods

### Study Design and Setting

This study is a follow-up study of the Marikina Memory and Aging Project (MMAP), which is a population-based cohort study to determine the prevalence of dementia and its associated risk among elderly in Marikina City, Philippines (6). Baseline assessment was done from March 2011 to February 2012 and follow-up assessment from July 2015 to December 2016. All follow-up assessments were conducted door-to-door. All participants provided written informed consent before any assessment was done.

### Participants

**Figure 1:**
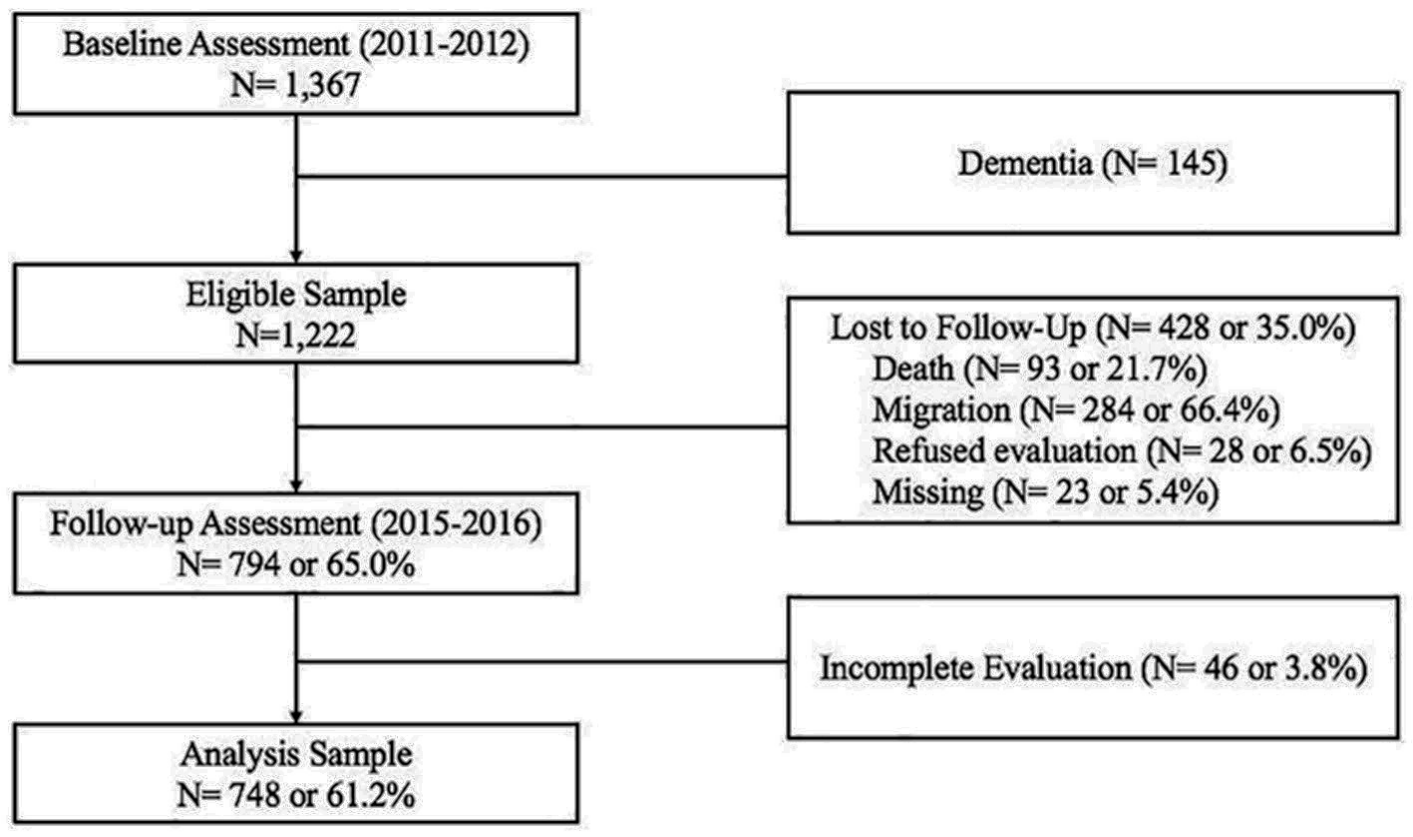
Flow chart of study participants.

The MMAP baseline cohort consists of participants randomly selected from the Senior Citizen Registry and is representative of percentages of senior citizens across villages (barangays) in the city of Marikina. The sampling method has been described previously (4). Recruitment was carried out door-to-door, and there were 1,367 participants who completed baseline assessment. For this incidence study, patients with dementia at baseline (*N* = 145) were excluded, leaving an eligible sample of 1,222. Of these, 794 participants (65.0%) were followed-up, among them 46 participants did not complete the evaluation and were excluded from subsequent analysis (Figure 1). All participants who were lost to follow-up were traced down to extract reasons for exclusion (as reported by neighbors and family members). The sample size for the current analysis was thus 748, with an average follow-up time of 3.9 years.

### Follow-Up Assessment and Case Ascertainment

Data collection, both at baseline and follow-up, used the same methodology of at-home visits, which included informant and patient interviews. Trained nurses gathered socio-demographic and health history information, and a trained psychologist administered the Montreal Cognitive Assessment-Philippines (MoCA-P) (7), Geriatric Depression Scale (GDS) (8), Neuropsychiatric Inventory-Questionnaire (NPIQ) (9), Disability Assessment for Dementia (DAD) (10), and Ascertaining Dementia 8 (AD-8) (11). A neurologist or geriatrician conducted medical history and physical examinations, Clinical Dementia Rating (CDR) (12), and Hachinski Ischemic Scale (HIS) (13). All assessment data were reviewed by the physician and they consulted with each other as necessary to reach a consensus on dementia diagnosis. Dementia diagnosis was based on the DSM-IV-TR criteria; (14) Alzheimer's Disease (AD) on the NINCDS-ADRDA criteria; (15) vascular dementia (VaD) based on the HIS. Other dementia subtypes such as Parkinson's disease dementia (16), Dementia with Lewy Bodies (17), and dementia syndrome of depression (14) were diagnosed based on clinical criteria. Cognitive Impairment No Dementia (CIND) was diagnosed based on a CDR score of 0.5 and not fulfilling DSM-IV criteria for dementia (18). These were the same criteria used during the baseline study. All baseline data for risk factors (i.e., vascular risks like hypertension, diabetes, dyslipidemia and smoking, alcohol consumption, traumatic brain injury, depression, and living arrangement) were collected from the participant or family informant.

### Disability-Adjusted Life Years Computation

Data from the study and relevant global literature were used to compute the DALYs using the DALY formula from the WHO. Number of deaths and mean duration of the disease were derived from dementia cases from the prevalence study. The disability weight used was 0.67 according to the WHO Global Burden of Disease in 2004 (19). Based on the Institute of Health Metrics and Evaluation Global Burden of Disease (20), average life expectancy at age 60 was 17 years irrespective of gender, 16 years for males, and 18 years for females. Since this is a global estimate, the researchers assumed that it applies to the Philippine context and also comparability to other similar studies. Note that the standard life expectancy at birth was not used since the population only included ≥60 years old. Life expectancy at age 60 years was the average number of years that a person at that age is expected to live if age-specific mortality levels remained constant.

### Cost of Care

Data was gathered from three groups of informants: (a) patients, relatives, and caregivers; (b) physicians including specialists (i.e., neurologist, geriatrician, and psychiatrist), non-specialists (i.e., family physician and internist), and allied health professionals (physical therapist, occupational therapist, psychologist), and (c) laboratories and drug stores. These groups were all interviewed in order to obtain information about fees and rates of the dementia patients' special needs.

### Data Analysis

Analysis was done using SPSS Statistical Program (IBM, ver. 27). Incidence rates per 1,000 person-years (pyr) were computed for each 10-year age interval and by sex. The corresponding 95% confidence intervals were computed based on the Exact Poisson distribution, as is standard practice in reporting incidence rates (21). Univariate statistics (accounts, means, SDs, ranges) and bivariate statistics (correlations) were produced. We assessed differences in socio-demographic variables and medical history related to dementia at follow-up status based on results from Chi-square tests and *t*-tests. Crude odds ratio was also presented to show the association of risk factors and incident dementia. All analyses were evaluated based on a statistical cutoff criterion for significance of 0.05. To compare incidence rates based on sex, rate ratios and their corresponding confidence intervals were calculated.

In terms of the Burden of Disease, the WHO DALY formula [DALY = Years of Life Lost (YLL) + Years Lost due to Disability (YLD)] was used. YLL is the number of deaths multiplied by the standard life expectancy. YLD is the number of incident cases multiplied by mean duration of disease multiplied by disability weight of AD and other dementias.

Cost of care was calculated by adding the direct and indirect costs. Direct medical cost was calculated as the sum of the costs, which included fees for consultations, laboratory assessments, hospital admissions or emergency visits due to problems related to dementia, dementia medications, and aids for mobility and rehabilitation. Direct non-medical costs refer to expense like transportation to and from the hospital for both the caretaker and the patient. Indirect cost used in this study refers to the opportunity cost calculated by the number of days the unpaid caregivers spent taking care of the dementia patient for one entire year multiplied by the daily minimum wage in Marikina City (Php 491.00) (USD 10.91) and then multiplied by the number of unpaid caregivers the patient had. Individual costs were computed per component and aggregated for the two major cost themes (direct vs. indirect). All samples were summarized using the median value.

### Ethics

The study was approved by the St. Luke's Institutional Ethics Review Committee (CT-14064).

## Results

### Dementia Incidence

The incidence rate was 16 (CI: 13–20) cases per 1,000 person-years (pyr). Participants with incident dementia were significantly older at baseline and had lower number of years of education. There was a higher proportion of baseline cognitive impairment (CIND) among those who developed dementia (Table 1). This sample mostly comprised females (73.3%). Among subtypes, the majority of cases were Alzheimer's disease (79%) followed by vascular dementia (18.5%) (Table 2). Dementia incidence was higher in females, with 17 (CI: 13–21) per 1,000 pyr compared to 14 (CI: 9–21) per 1,000 pyr for males (Table 3). Incidence rates increased with age, except for in males age 80 years and above.

**Table 1:** Baseline profile of participants with and without incident dementia.

**Baseline characteristics**	***N***	**Incident dementia (*N* = 81)**	**No dementia (*N* = 667)**	**Total (*N* = 748)**	***p*-value**	OR^*^	**95% CI**
Age (years, M ± SD; min-max)	748	73.8 ± 8.0 (60.0–99.0)	68.7 ± 5.9 (59.0–90.0)	69.2 ± 6.4	<0.001	N/A	N/A
Years of education (M ± SD)	748	6.1 ± 3.6	8.9 ± 3.8	8.6 ± 3.9	<0.001	N/A	N/A
Male (%)	748	21 (25.9)	179 (26.8)	200 (26.7)	0.884	0.962	0.57–1.63
Female (%)		60 (74.0)	488 (73.2)	548 (73.3)			
With cognitive impairment (*N*, %)	748	39 (48.1)	155 (23.2)	194 (25.9)	<0.001	3.067	1.91–4.92
With vascular risks^δ^ (*N*, %)	743	62 (76.5)	470 (71.0)	532 (71.6)	0.296	1.333	0.78–2.29
With hypertension (*N*, %)	711	49 (62.8)	346 (54.7)	395 (55.6)	0.171	1.402	0.86–2.28
With dyslipidemia (*N*, %)	600	16 (25.8)	189 (35.1)	205 (34.2)	0.143	0.642	0.35–1.17
With diabetes (*N*, %)	694	14 (18.2)	107 (17.3)	121 (17.4)	0.855	1.059	0.57–1.96
With smoking history (*N*, %)	735	22 (27.2)	137 (20.9)	159 (21.6)	0.200	1.407	0.83–2.38
With alcohol abuse history (*N*, %)	717	14 (17.5)	112 (17.6)	126 (17.6)	0.985	0.994	0.54–1.83
With TBI^δδ^ (*N*, %)	715	2 (2.5)	10 (1.6)	12 (1.7)	0.531	1.626	0.35–7.56
With depression history (*N*, %)	712	36 (44.4)	276 (43.7)	312 (43.8)	0.904	1.029	0.65–1.64
Living alone (*N*, %)	748	5 (6.2)	31 (4.6)	36 (4.8)	0.545	1.35	0.51–3.58
Stroke (*N*, %)	718	9 (11.3)	41 (6.4)	50 (7.0)	0.109	1.849	0.86–3.96
Heart disease^**^ (*N*, %)	726	26 (4.9)	8 (4.2)	34 (4.7)	1.000	0.762	0.23–2.35
Reported physical difficulty^δδδ^ (*N*, %)	748	36 (44.4)	151 (22.6)	187 (25.0)	<0.001	2.734	1.70–4.39

*Data presented as mean ± standard deviation (SD) or frequency of present risk factors with percentage in the parentheses and p-values obtained using t-tests for independent samples or Pearson's Chi-Square test/Fisher's Exact Test*.

* *Crude associations and odds ratios are presented*.

** *Heart disease includes at least one of the following conditions: heart attack, atrial fibrillation, endarterectomy, bypass, pacemaker, and congestive heart disease*.

δ *Vascular risks include at least one of the following: hypertension, dyslipidemia, diabetes and smoking history*.

δδ *TBI means traumatic brain injury which may include TBI with brief or extended loss consciousness*.

δδδ *One or more reported physical difficulty in activities of daily living requiring assistance from family*.

**Table 2:** Diagnosis of dementia subtypes in the incident cases.

**Diagnosis**	**Total (%)**	**Male; *N* (%)**	**Female; *N* (%)**	***p*-value**
Alzheimer's disease (AD)	64 (79.0)	12 (57.1)	52 (86.7)	
Vascular dementia (VaD)	15 (18.5)	8 (38.1)	7 (11.7)	
Parkinson's Disease Dementia (PDD)	1 (1.2)	0 (0.0)	1 (1.7)	0.01
Dementia syndrome of depression	1 (1.2)	1 (4.8)	0 (0.0)	
Total	81 (100.0)	21 (100.0)	60 (100.0)	

*p-value obtained using Chi-square test for comparison of proportions*.

**Table 3:** Age and gender-specific incidence rate for dementia (per 1,000 person-years).

**Age (in years)**	Total (^*^95% CI)	Female (^*^95% CI)	Male (^*^95% CI)	Rate ratio (^*^95% CI)
60–69	9.13 (5.99–13.38)	7.67 (4.37–12.56)	12.46 (6.33–22.21)	0.62 (0.25–1.55)
70–79	18.78 (13.48–25.52)	19.06 (12.91–27.18)	18.05 (9.17–32.18)	1.06 (0.50–2.44)
80 and above	41.82 (25.92–64.10)	54.80 (33.5–84.94)	7.94 (0.39–39.18)	6.90 (1.09–287.47)
Total	15.86 (12.68–19.62)	16.56 (12.75–21.17)	14.17 (9.00–21.29)	1.16 (0.70–2.02)

* *Confidence intervals based on an Exact Poisson Distribution are shown in parentheses*.

### Disability Adjusted Life Years

Mean total disease duration was 3.84 years (3.85 years for males and 3.83 for females). There were 24 recorded deaths, with 14 male and 10 female decedents. Using this number of deaths and the standard life expectancy for Filipinos, years of life lost (YLL) was calculated as 404 years (224 years for males and 180 years for females). Using the incident cases, duration of disease, and disability weights, the years lost due to disability (YLD) was 208 (54 for males and 154 for females). From this information, crude DALYs was estimated as 2,876 years per 100,000 persons, 4,891 per 100,000 persons for males and 2,142 per 100,000 persons for females (Table 4).

**Table 4:** Data for the computation of disability-adjusted life years.

**Variables**	**Total**	**Male**	**Female**
Marikina city seniors population (2015)^*^	21,275	5,684	15,591
Incident cases	81	21	60
Disability weights^***^	0.67	0.67	0.67
Average (SD) disease duration (in years)^**^	3.84 (0.30)	3.85 (0.30)	3.83 (0.31)
Number of deaths^**^	24	14	10
Life expectancy at 60(years)^****^	17	16	18
Years of life lost^δ^	404	224	180
Years lost due to disability^δδ^	208	54	154
DALY	612	278	334
DALY ('00000)^δδδ^	2,876	4,891	2,142

* *Total size was obtained from Marikina City records, male/female distribution was estimated using sample proportions*.

** *Obtained from this incidence cohort*.

*** *As per World Health Organization Global Burden of Disease*.

**** *As per Institute of Health Metrics*.

δ *Computed as the product of number of deaths and the standard life expectancy at the age of death*.

δδ *Computed as the product of incident cases by duration and disability weight of the condition*.

δδδ DALY ('00000) is [(YLL+YLD)/population size] ^*^ 100,000.

### Cost of Care

The median direct medical cost for dementia care was Php 11,419.03 (USD 237.40) while direct non-medical cost was Php 316.00 (USD 6.57). Total median direct cost was therefore Php 11,016.50 (USD 229.03). Calculating for annual indirect costs accumulated through unpaid caregiving, the median indirect cost was Php 175,565.00 (USD 3,650). The sum of direct and indirect costs (total costs) was Php 188,381.98 (USD 3,916.47) per capita annually (Table 5). The majority (93.2%) of this care expense was indirect cost attributed to estimated lost potential earning of unpaid family caregivers whereas direct medical cost accounted for only 6.8%.

**Table 5:** Cost of care of community-based patients with dementia.

**Type of cost**	Median (in USD^*^)	25th PCT (in USD^*^)	75th PCT (in USD^*^)	Minimum (in USD^*^)	Maximum (in USD^*^)
Aggregated direct costs^a^	229.03	62.98	546.09	0.15	2,999.53
Direct medical^b^	237.40	109.08	729.93	0.15	2,999.53
Direct non- medical costs^c^	6.56	3.07	16.32	1.16	28.07
Indirect costs^d^	3,650.00	3,650.00	3,650.00	0.8	18,250.00
Overall costs^e^	3,916.47	1,175.75	4,125.68	30.45	379.42

a *Includes Medical and Non-medical cost*.

b *Includes salary of caregiver; special diets; drugs; hospitalization costs; and rehabilitative aids*.

c *Includes transportation costs to health facilities*.

d *Potential earning capacity of unpaid caregivers*.

e *Aggregate of direct and indirect costs*.

*Individual costs were computed per component, aggregated for the two major cost themes (direct and indirect) then all samples were summarized using median*.

* *48.1 Php = 1 USD (November 2020)*.

### Representativeness and Projection

The cohort is demographically representative of senior citizens in the Philippines with respect to age (mean age of 70 years) and years of education (mean of 8.5 years) at study inception in 2015 (14). Geographically, it is representative of Filipino older persons residing in urban and semi-rural areas. The division of urban and rural dwelling in the Philippines was ~50–50 in 2019 (22).

Dementia incidence was 16 cases per 1,000 pyr (95% CI: 12.7–19.6). Using the calculated incidence rate and projected senior citizens population, the Philippines has 149,606 new dementia cases in 2020 and will have 220,632 in 2030, 295,066 in 2040, and 378,461 in 2050 (23) (see Supplementary Table 1a).

## Discussion

This is the first large longitudinal community-based cohort study in the Philippines, first to establish prevalence and subsequently incidence of dementia, providing the first evidence-based data on disease-occurrence and burden. It is a wake- up call for policy makers, as dementia prevalence in the Philippines was shown to be the double of the estimate for South East Asia by World Alzheimer Report 2015. Furthermore, the current study showed that dementia incidence in the Philippines, at 16 per 1,000 pyr, is as high as in East Asia, where studies have shown an incidence rate of 13.50/1,000 pyr (1). This is close to the reported 14.06 per 1,000 pyr of the World Alzheimer Report (1) for LMIC. The projected number of new cases for 2020 in the Philippines makes up one quarter of the annual estimate for the whole South East Asia (1), and that number will increase by 50% in 2030 and 100% in 2040 (see Supplementary Table 1a). Using the prevalence rate derived from this cohort in 2011, the Philippines is projected to have total of 1,474,588 dementia cases in 2030, 1,972,067 in 2040 and 2,529,436 in 2050 (see Supplementary Table 1b). Clearly, this rapid and marked increase in dementia occurrence will pose a formidable burden on the healthcare system and the financial capacities of affected households. Currently, the Philippines has no social and health policies to address the dementia epidemic. In order to avoid an impending public health crisis, social and health care policy reform is urgently needed.

Consistent with the global trend, dementia incidence in this cohort increased with age, doubling at every 10-year age group. There was no gender-based difference in dementia incidence among younger participants (60–79 years old), but there was a gender difference in dementia incidence among older participants (80 and above). This is consistent with results from a large population cohort from the Netherlands where a striking decrease in incidence was also seen in males aged above 89 and females were more at-risk to develop dementia by 2.61 times (24). Such trends in women across the age groups (i.e., increasing over the years) and the shifting of males (i.e., more cases in the younger age groups then declining) were also present in a community-dwelling Brazilian cohort (25). In a systematic review for dementia studies across the globe on individuals 60 and over residing in the community, the pooled incidence rate (same age and setting) was 17.18 (95%: 13.90–21.23) per 1,000 person-years with estimates for females consistently higher than males (26).

Aside from age, we found lower years of education, presence of cognitive impairment, and reported difficulty with physical function at baseline to be associated with higher incident dementia. Low education is a risk factor with high weighted population attributable fraction (PAF) of dementia and an important target for dementia prevention intervention (27). In this cohort, disparity in education disproportionately affected females (see Supplementary Table 2). These latter results indicate that efforts to make early life education equally accessible to all citizens (up to at least a high school diploma, which was recently increased from 10 to 12 years of formal education in the Philippines) may be especially important for dementia prevention (28). Mean follow-up time of 3.9 years is similar to follow-up times from prior cohort studies from high-income countries (29).

Two thirds of the cohort had at least one vascular risk, with males having significantly more, e.g., higher number of strokes, smoking, and alcohol abuse (see Supplementary Table 2), consistent with the country's profile. These modifiable lifestyle-related factors such as smoking and alcohol drinking were seen to have gender-based differences since males were more likely to have such behaviors (30, 31). However, we did not find baseline vascular risks, stroke, or heart disease to be associated with incident dementia. In this cohort, incident vascular dementia—for which vascular risks, stroke and heart disease are relevant in terms of dementia pathology—was only 18%. This is likely due to the high mortality rate associated with stroke in the Philippines (32). On the other hand, it may also be hypothesized that vascular risks are managed well as Marikina is one of the cities that has a strong health program for its senior citizens. This can be verified in a future follow-up of the cohort.

With improving care for acute stroke, we expect to see more survivors and likely vascular dementia in younger and working populations <60 years old in the future. The resulting disability will further contribute to increased dementia burden in the population. When incident cases, duration of disease, and disability weights were taken into account in our cohort, the years lost due to disability was 208 and the crude DALY rate was computed to be 2,876 per 100,000 persons (Table 3). Using this rate, our calculated DALY was 219,706. This is close to the estimated Philippine DALY value of 230,296 by the Global Burden of Disease (GBD) study 2016 (33). This is double of the estimated DALY for European countries (18). Dementias rank second in the global burden of disease among neurological disorders (34). Traditionally, health statistics and epidemiology place emphasis on mortality rather than disability of a disease entity, resulting in an underestimation of the burden of disabling neurological conditions such as dementia (35). The long-term and progressive disability from dementia is further complicated by a high economic burden in both formal and informal care, largely attributed to unpaid family caregivers in LMIC and to lost opportunity for participation in the labor force. Ironically, there is limited data on the economic costs of dementia in LMIC. This may be due to low priority given to mental health, an absence of trained health economists, and/or inadequate development of mental health services (36). This study revealed that the cost of informal care in the Philippines is exceptionally high, making up 86% of total cost, reflecting the enormous economic strain on the Filipino families. In comparison, the average estimated informal care cost for LMIC is ~60% (1).

The current healthcare provision system in the Philippines is decentralized, with the Department of Health (DOH) serving as the governing agency and private sectors and local government units providing services to communities (37). The country's national health insurance program, Philippine Health Insurance Corporation (PhilHealth), and other health provision systems such as the Social Security System (SSS) cover for direct medical costs only during hospital admissions (38, 39). Even with government social welfare programs and compensations for medical costs, the affordability of Alzheimer's disease medications remains a challenge in the LMICs. With an affordability index (total number of units purchasable by one's daily income) of 0.8 tablet in the Philippines (40), national health insurance makes little difference in overall economic burden for affected individuals and families. The burden goes beyond direct medical costs because a large proportion of dementia care involves long-term residential care which results in informal costs exceeding formal costs. In our data, the average direct medical cost accounted for only 13.48% while indirect costs of care accounted for 86.29% (Supplementary Table 3). The median, minimum and maximum costs are presented in Table 5.

Moreover, direct medical cost was undoubtedly underestimated given that the majority of dementia cases in the cohort were diagnosed during the study and were not treated with anti-dementia drugs. There were no dementia-specific support services in place in the community for patients to access. Direct cost was related mainly to emergency hospitalization due to dementia-associated events such as falls, infections, and behavioral disturbances. As noted, overall cost of care was mainly attributed to indirect expenses. A major reason for this is that in the Philippines (and in many other LMIC), informal healthcare cost is driven by cultural factors, such as filial piety and obligatory care (41, 42) which removes family caretakers from the workforce. This is consistent with findings in other neighboring Asian countries where a family member cares for the patient with dementia (92.4% in the Philippines) (43). Indirect cost is further underestimated for caretakers who are skilled workers and earning higher than minimum wage (i.e., >USD 10.9/day) and who are unable to work while providing care. Thus, the burden appears to be less on the formal healthcare system per se and more on the financial capacities of households. The data presented in our study therefore calls for an urgent need for government efforts to develop a national dementia care plan in line with recommendations of the World Health Organization to increase subsidy for direct costs and include social benefits and formal training for family caregivers.

Although some dementia prediction models developed in high-income countries may be extrapolated to developing countries, country-specific primary data is still very much needed in LMIC (44). This is a key contribution of our study which provides direct, instead of extrapolated, dementia incidence data. This is further strengthened by its use of repeated, longitudinal, and multi-modal assessments (participants were evaluated by a neurologist or geriatrician, and family informants were also interviewed).

## Limitations

One limitation in this study is that about 35% (*N* = 475) of the eligible sample were lost to follow-up or were not included in the analysis due to incomplete data, similar to other LMIC cohorts (45). The main reason was mass migration from the city due to consecutive years of frequent flooding between 2012 and 2014. Nevertheless, analysis for selection bias showed insignificant differences between participants with and without follow-up in terms of age, education and cognitive impairment. Possible bias was only seen in terms of sex as the cohort in the baseline study was proportionally allocated based on residence. Since the subject population in this study is a follow-up of the baseline cohort, it should be noted that mostly males were lost to follow-up (Supplementary Tables 4a,b), resulting in the disparate sex distribution reported. Another limitation in this study is the lack of neuroimaging mainly due to high costs in a community-setting of such a sample size. In terms of DALY computation, there are no local data in the Philippines. The estimated life expectancy was therefore derived from the Institute of Health Metrics. Furthermore, there were also no DALY estimates for individual patients, hence we were unable to estimate the 95% confidence interval. For this study, we assumed that the global estimate is applicable to our setting. Lastly, since this is the first community-based cohort study of older adults in the Philippines, the population included was comparatively smaller with fewer risk factors examined than in other 10/66 Dementia Research Group LMIC cohorts. For future follow-up of the cohort, more recently identified risk factors targeted for dementia prevention, such as air pollution (27), a serious problem in LIMCs, will be taken into consideration.

## Conclusion

We provide the first local community-based data on the incidence of dementia and dementia-related disability adjusted life years and cost of care in the Philippines. Dementia incidence and cost of care was close to estimates in other LMIC. Since informal (family) care is the cornerstone of dementia care in the Philippines, and since educational disparities appeared as a key risk factor for dementia, effective government initiatives for dementia prevention and care should target childhood education for all and assistance for family caregivers. Given the constraints of resource allocation in the LMIC as well as lack of programs geared toward cognitive decline and management of modifiable risk factors for the population of older adults, this study provides data for evidence-based healthcare planning in line with WHO recommendations.

## Data Availability Statement

The raw data supporting the conclusions of this article will be made available by the authors, without undue reservation.

## Ethics Statement

The studies involving human participants were reviewed and approved by St. Luke's Institutional Ethics Review Committee. The patients/participants provided their written informed consent to participate in this study.

## Author Contributions

JaD and LJ conceptualized the research and methodology and acquired funding. JaD, LJ, MD, JD-A, BN, JeD, and JDD were responsible for data collection. KF, MR, and AL conducted the analysis. JaD, KF, and JY wrote the original draft of the manuscript. SA and TP supervised analysis and manuscript organization. All authors contributed to reviewing and editing the manuscript.

## Conflict of Interest

The authors declare that the research was conducted in the absence of any commercial or financial relationships that could be construed as a potential conflict of interest.
