# Supplementary material for: Dementia Incidence, Burden and Cost of Care: A Filipino Community-Based Study
Source: Front Public Health. 2021 May 14;9:628700. doi: 10.3389/fpubh.2021.628700 (PMC8160123; doi:10.3389/fpubh.2021.628700)
Supplement: Supplementary file 1 [file Table_1.DOCX]

**SUPPLEMENTARY MATERIALS**

**Table 1a.** Incident Dementia Projections (2020-2050) based on 2015 Incidence Rate

| Age Group | Incidence Rate per 1000 PY | Incidence Prop | Projected Population (2020) | Projected Population (2030) | Projected Population (2040) | Projected Population (2050) | Projected Incident Cases (2020) | Projected Incident Cases (2030) | Projected Incident Cases (2040) | Projected Incident Cases (2050) |
| --- | --- | --- | --- | --- | --- | --- | --- | --- | --- | --- |
| 60-69 | 9.13 | 0.00913 | 5,878,871 | 8,062,777 | 9,982,021 | 12,365,881 | 53,674 | 73,613 | 91,136 | 112,900 |
| 70-79 | 18.78 | 0.01878 | 2,639,619 | 4,361,189 | 6,094,963 | 7,714,047 | 49,572 | 81,903 | 114,463 | 144,870 |
| 80 and above | 41.82 | 0.04182 | 914,418 | 1,487,245 | 2,527,421 | 3,782,675 | 38,241 | 62,197 | 105,697 | 158,191 |
| Total | 15.86 | 0.01586 | 9,432,908 | 13,911,211 | 18,604,405 | 23,862,603 | 149,606 | 220,632 | 295,066 | 378,461 |

**Table 1b.** Prevalent Dementia Projections (2020-2050) based on 2011 Prevalence Rate

| Age Group | Prevalence | Projected Population (2020) | Projected Population (2030) | Projected Population (2040) | Projected Population  (2050) | Projected Prevalent Cases (2020) | Projected Prevalent Cases (2030) | Projected Prevalent Cases (2040) | Projected Prevalent Cases (2050) |
| --- | --- | --- | --- | --- | --- | --- | --- | --- | --- |
| 60-69 | 5.9% | 5,878,871 | 8,062,777 | 9,982,021 | 12,365,881 | 346,853 | 475,704 | 588,939 | 729,587 |
| 70-79 | 13.6% | 2,639,619 | 4,361,189 | 6,094,963 | 7,714,047 | 358,988 | 593,122 | 828,915 | 1,049,110 |
| 80 and above | 23.6% | 914,418 | 1,487,245 | 2,527,421 | 3,782,675 | 215,803 | 350,990 | 596,471 | 892,711 |
| Total | 10.6% | 9,432,908 | 13,911,211 | 18,604,405 | 23, 862,603 | 999,888 | 1,474,588 | 1,972,067 | 2,529,436 |

**Table 2.** Baseline Demographics and Risk Characteristics of Study Participants

| **Characteristics at baseline** | **N** | **Male**  **(N = 199)** | **Female**  **(N = 549)** | **Total**  **(N = 748)** | **p-value** |
| --- | --- | --- | --- | --- | --- |
| Age | 748 | 68.7 ± 5.9 | 69.4 ± 6.5 | 69.2 ± 6.4 | 0.194 |
| Years of Education | 748 | 9.8 ± 3.5 | 8.2 ± 3.9 | 8.6 ± 3.9 | < 0.001 |
|  |  |  |  |  |  |
| With Cognitive impairment | 748 | 56 (28.1) | 138 (25.1) | 194 (25.9) | 0.407 |
| Vascular Risk Score^*^ | 743 | 2.0 ± 1.2 | 1.3 ± 1.2 | 1.5 ± 1.2 | < 0.001 |
| Stroke | 719 | 22 (11.6) | 28 (5.3) | 50 (7.0) | 0.003 |
| Heart Disease^**^ | 726 | 8 (4.2) | 26 (4.9) | 34 (4.7) | 0.706 |
| Vascular Risk factor ^δ^ | 743 | 157 (79.3) | 375 (68.8) | 532 (71.6) | 0.005 |
| Hypertension | 711 | 97 (52.7) | 298 (56.5) | 395 (55.6) | 0.368 |
| Dyslipidemia | 600 | 36 (23.8) | 169 (37.6) | 205 (34.2) | 0.002 |
| Diabetes | 694 | 28 (15.6) | 93 (18.1) | 121 (17.4) | 0.463 |
| Smoking history | 735 | 107 (54.3) | 52 (9.7) | 159 (21.6) | < 0.001 |
| Alcohol abuse | 717 | 92 (48.4) | 34 (6.5) | 126 (17.6) | < 0.001 |
| TBI ^δδ^ | 715 | 3 (1.6) | 9 (1.7) | 12 (1.7) | 1.00 |
| Depression history | 712 | 65 (34.2) | 247 (47.3) | 312 (43.8) | 0.002 |
| Living Alone | 748 | 4 (2.0) | 32 (5.8) | 36 (4.8) | 0.031 |
| Reported Physical Difficulty^δδδ^ | 748 | 44 (22.1) | 143 (26.0) | 187 (25.0) | 0.272 |
| Data presented as mean ± standard deviation (SD) or frequency of present risk factors with percentage in the parenthesis;  p-values obtained using t-test for independent samples or Pearson’s chi-square test;  ^*^ Vascular risk score includes at least one of the following conditions: smoking history, alcohol abuse, diabetes, dyslipidemia, hypertension, stroke, and heart disease.  ^**^ Heart disease includes at least one of the following conditions: heart attack, atrial fibrillation, endarterectomy, bypass, pacemaker, and congestive heart disease;  ^δ^ Vascular risk factor includes at least one of the following: hypertension, dyslipidemia, diabetes and smoking history;  ^δδ^ TBI means traumatic brain injury which may include TBI with brief or extended loss consciousness  ^δδδ^ One or more reported physical difficulty in activities of daily living requiring assistance from family | | | | | |

**Table 3.** Cost of Care of Community-Based Patients with Dementia

| **Type of Cost** | Average Cost (in USD*) ± SD | Lower limit (in USD*) | Upper limit (in USD*) | Minimum (in USD*) | Maximum (in USD*) | % of Total Cost |
| --- | --- | --- | --- | --- | --- | --- |
| **Total Direct Costs**^1^ | 547.66 ± 686.11 | 385.58 | 729.93] | 1.31 | 3,027.30 | 13.71% |
| Direct Medical^2^ | 548.51± 678.15 | 378.33 | 718.69 | 0.15 | 2,999.23 | 13.48% |
| Direct Non- Medical Costs^3^ | 9.25 ± 7.96 | 7.25 | 11.25 | 1.16 | 28.06 | 0.22% |
| **Indirect Costs**^4^ | 3,510.38 ± 576.53 | 3,365.70 | 3,655.06 | 479.95 | 3,793.81 | 86.29% |
| **Total Costs**^5^ | 4,068.14 ± 1262.64 | 3,751.28 | 4,384.99 | 481.27 | 6,821.11 | 100.00% |
| ^1^Includes Medical and Non-medical cost; ^2^Includes salary of caregiver; special diets; drugs; hospitalization costs; and rehabilitative aids; ^3^Includes transportation costs to health facilities; ^4^Potential earning capacity of unpaid caregivers; ^5^Sum of direct and indirect costs  *48.1Php= 1USD (November 2020) | | | | | | |

**Table 4a.** Age and education of participants lost to follow-up compared to follow-up

| Baseline characteristic | No Follow-Up | | | Follow-Up | | | Total | | | |
| --- | --- | --- | --- | --- | --- | --- | --- | --- | --- | --- |
|  | N | Mean | SD | N | Mean | SD | N | Mean | SD | p-value |
| Age (years) | 474 | 69.81 | 6.91 | 748 | 69.21 | 6.39 | 1222 | 69.45 | 6.6 | 0.121 |
| Education (years) | 474 | 8.93 | 3.98 | 748 | 8.63 | 3.86 | 1222 | 8.74 | 3.91 | 0.187 |

**Table 4b.** Sex and cognitive impairment among participants lost to follow-up compared to followed-up

| Baseline characteristic | | No Follow-Up | | Follow-Up | | Total | |  |
| --- | --- | --- | --- | --- | --- | --- | --- | --- |
|  |  | N | % | N | % | N | % | p-value |
| Sex | Male | 157 | 44.10% | 199 | 55.90% | 356 | 29.10% | 0.016 |
|  | Female | 318 | 36.70% | 548 | 63.30% | 866 | 70.90% |  |
| Cognitive impairment | Positive | 123 | 38.80% | 194 | 61.20% | 317 | 25.90% | 0.976 |
|  | Negative | 352 | 38.90% | 553 | 61.10% | 905 | 74.10% |  |
